# COVID-19 Community Testing In Rural Areas: A Partnership between an Academic Medical Center and Community Clinics

**Published:** 2020-07-10

**Authors:** T Davis, J Singh, JG Lance, L Latiolais, C Kevil, J Bodily, M Sapp, R Scott, P Weinberger, J Vanchiere, C Arnold

**Affiliations:** 1 Department of Medicine, LSU Health - Shreveport, Shreveport, Louisiana, USA; 2 Department of Microbiology and Immunology, LSU Health - Shreveport, Shreveport, Louisiana, USA; 3 Department of Pathology, School of Graduate Studies, LSU Health - Shreveport, Shreveport, Louisiana, USA; 4 Department of Otolaryngology, LSU Health - Shreveport, Shreveport, Louisiana, USA; 5 Department of Pediatrics, LSU Health - Shreveport, Shreveport, Louisiana, USA

**Keywords:** Access to COVID-19 screening, Health disparities, Health services research, Community clinics, Mobile health

## Short Communication

The coronavirus pandemic is shedding light on existing health disparities and has the potential to accentuate both racial and rural/urban health divides [1]. The pandemic that first struck in major metropolitan areas is increasingly findings its front line in rural counties [2]. The Kaiser Family Foundation recently reported that coronavirus cases and deaths are surging at a faster rate in rural areas compared to urban areas. Also of concern, rural areas without confirmed cases may lack testing necessary for an accurate assessment of virus prevalence. Expanding access to reliable testing in isolated rural areas is an immediate challenge.

Rural residents are at high risk for poor health outcomes with COVID-19 due to being older, poorer and more likely to be underinsured compared to urban counterparts [3–5]. Rural populations also have higher rates of chronic health conditions such as hypertension, obesity, diabetes, coronary heart disease, and cigarette smoking that increase the risk of severe COVID-19 [3,5]. Moreover, rural areas often lack hospitals to serve as screening/testing coordinating sites, and to provide specialist capacity to take care of severely ill patients with the virus [6–7].

Our medical center has a mission to serve vulnerable populations in our state and to help address health disparities. When the coronavirus began to rapidly spread in Louisiana (LA) we responded to state and regional concerns about lack of rapid, reliable testing in many rural parishes in our area. We partnered with rural primary care clinics to rapidly provide trusted testing and timely return of results. Louisiana State University Health System-Shreveport (LSUHS-S) has a concentration of expertise in molecular analysis, as well as equipment and facilities suitable for adapting to responses to new pathogens. We developed viral tests, independent of major supply chains, and had access to adequate supplies, enabling us to make testing kits. Our partnership with rural Federally Qualified Health Centers (FQHCs) allowed more rapid and reliable COVID-19 testing in isolated rural communities. FQHCs are community-based health centers that provide primary care in underserved areas to our nation’s most vulnerable populations. One in 5 rural residents and 1 in 3 individuals living in poverty receive care at FQHCs. As states relax social distancing restrictions implemented due to COVID-19, these partnerships can also facilitate rural participation in clinical trials for antibody testing and vaccines.

The purpose of this commentary is to describe an innovative methodology to improve access to testing that was successfully implemented in nine rural parishes in North Louisiana. The IRB determined this project to be “Not human subject research.”

Our academic center’s rural community outreach testing began in April 2020 after the creation of the LSUHS-S Emerging Viral Threat (EVT) Lab. The EVT lab was designed to address the need for fast robust detection of COVID-19 cases and rapid processing of COVID-19 tests. LSUHS-S has an unusually high number of world-class virologists, therefore the human and intellectual resources needed to create a lab were already present. In addition to providing rapid detection of COVID-19 cases, the EVT lab developed the infrastructure necessary to assemble testing kits. This ability to assemble and distribute testing kits in substantial numbers eliminated one common barrier in rural areas of scarcity of testing supplies.

The initial steps of the rural outreach included health services research faculty (TD and CA) reaching out to FQHCs with whom they have trusted relationships based on previous health research and service collaborations. They called clinic administrators and medical directors to discuss screening needs and interest in utilizing the new EVT lab’s capacity to provide testing services and clinical research nurses trained to obtain nasopharyngeal swabs from patients. Our mobile community outreach mammography screening van was repurposed to facilitate testing outreach. The van transported testing kits and nurses to collect and transport COVID-19 testing. The mobile mammography program director and staff had a long history of providing cancer screening outreach to these same community clinics, which enhanced the brisk implementation and community trust of the mobile testing effort. The mobile outreach approach was appealing to the clinics, as the van provided visible public relations and promotion of the partnerships. The testing events, which usually lasted 2–4 hours, allowed patients to stay in their vehicles for registration and testing. Arrangements were also made to allow for walk-up patients who did not have a vehicle, further enhancing accessibility.

The COVID-19 outreach screening collaboration was aimed at meeting the needs of each clinic and the rural area they serve. Conference calls between the clinics and academic medical center faculty were held to work out details including anticipating potential problems involved for an efficient COVID-19 drive-through-testing event. The collaborative plan included advertising, prescreening patients, traffic management and security, collection of specimens, and distribution of test results. The FQHCs handled advertising and prescreening patients. They coordinated with local officials to determine the time, date, and location of each COVID-19 mobile testing event and to provide traffic management and safety. Each clinic advertised through local media, social media, and flyers. The clinics created short registration and consent forms, and clinic providers or medical assistants prescreened patients to confirm eligibility for testing according to CDC guidelines. The clinic providers placed the order for the test for each patient, and the results were sent back to each clinic which subsequently notified patients of their results. In cases where an individual did not have a primary care provider, the FQHC agreed to be the provider of record, order the test, receive the results, and notify the patient.

Brief COVID -19 plain language education was developed by our team and provided to individuals who were tested. The colorful one-page handout was written on a 6th grade level, formatted for reading ease and illustrated with simplified CDC graphics that emphasized key points. Before leaving the testing site, each patient received an educational handout in English or if appropriate, in Spanish and a “quarantine kit”. The instructional handout focused on what individuals’ and families needed to know and do after being tested. It had step-by-step instructions on how to self-quarantine if needed, when to call their healthcare provider or 911 and reinforced basic safety steps to prevent the spread of COVID-19. The “quarantine kit” was a zip-top bag containing two cloth or surgical masks, a bottle of hand sanitizer, and hand soap with materials being sourced locally, to facilitate adherence to recommendations. There was no charge associated with testing, or for the “quarantine kit.” For any patients requiring follow-up care because of an illness or positive viral test result, normal clinic charges would apply for the provider delivering that care.

To date we have tested 431 individuals in rural communities with populations ranging from 526–13,000. These communities were located 30–131 miles from the medical center. The individuals tested ranged from 24 months to 96 years of age (median 53 years), 62.2% were African American, 36.3% white, and 0.2% American Indian. The majority of patients tested (69.6%) were female. Nine (2.1%) of the individuals screened had a positive test result and have been followed-up by their FQHC primary care provider.

## Conclusions

Expanding access to reliable screening and establishing trust among rural populations is critical to containment efforts for COVID-19. Academic medical center and rural FQHC collaboration, bidirectional planning, and coordinated implementation enabled a well-organized screening in rural communities. National Guard teams, local law enforcement, and parish and city officials helped ensure organization and safety. These COVID-19 testing relationships with rural primary care and school-based clinics likely to continue with the need for viral genome surveillance and antibody testing for detection of previous infection. These relationships may also facilitate participation of rural patients in COVID-19 related clinical trials [8].
